# ClineHelpR: an R package for genomic cline outlier detection and visualization

**DOI:** 10.1186/s12859-021-04423-x

**Published:** 2021-10-16

**Authors:** Bradley T. Martin, Tyler K. Chafin, Marlis R. Douglas, Michael E. Douglas

**Affiliations:** 1 Arkansas Conservation and Molecular Ecology Laboratory, Department of Biological Sciences, University of Arkansas, Fayetteville, AR USA; 2 University of Arkansas Global Campus, Fayetteville, AR 72701 USA; 3 Ecology and Evolutionary Biology Department, University of Colorado, Boulder, CO USA

**Keywords:** Hybrid zones, bgc, Introgression, Population genetics, Selection, Outlier detection, Genomic cline

## Abstract

**Background:**

Patterns of multi-locus differentiation (i.e., genomic clines) often extend broadly across hybrid zones and their quantification can help diagnose how species boundaries are shaped by adaptive processes, both intrinsic and extrinsic. In this sense, the transitioning of loci across admixed individuals can be contrasted as a function of the genome-wide trend, in turn allowing an expansion of clinal theory across a much wider array of biodiversity. However, computational tools that serve to interpret and consequently visualize ‘genomic clines’ are limited, and users must often write custom, relatively complex code to do so.

**Results:**

Here, we introduce the ClineHelpR R-package for visualizing genomic clines and detecting outlier loci using output generated by two popular software packages, bgc and Introgress. ClineHelpR bundles both input generation (i.e., filtering datasets and creating specialized file formats) and output processing (e.g., MCMC thinning and burn-in) with functions that directly facilitate interpretation and hypothesis testing. Tools are also provided for post-hoc analyses that interface with external packages such as ENMeval and RIdeogram.

**Conclusions:**

Our package increases the reproducibility and accessibility of genomic cline methods, thus allowing an expanded user base and promoting these methods as mechanisms to address diverse evolutionary questions in both model and non-model organisms. Furthermore, the ClineHelpR extended functionality can evaluate genomic clines in the context of spatial and environmental features, allowing users to explore underlying processes potentially contributing to the observed patterns and helping facilitate effective conservation management strategies.

## Background

Patterns of multi-locus differentiation, as distributed across admixture gradients, have long provided a window into divergence and speciation [e.g., 1, 2]. Accordingly, they have been used to map loci associated with adaptation or reproductive isolation [3, 4], and as indicators of biotic responses to environmental change [5]. Rather than relating these to patterns in the landscape, contemporary approaches have instead drawn conclusions based on genome-wide ancestries [6, 7]. The evolutionary processes that generate ‘genomic clines’ can be illuminated even when constituent taxa do not segregate geographically, but rather patchily [8], or as a hybrid mosaic [5].

Several programs are available specifically to investigate genomic clines. Of these, bgc [9, 10] is the most robust to false positives and uses a Bayesian approach that accounts for genotype uncertainty [11] and autocorrelation caused by physical linkage [12] in next-generation sequencing datasets. To compliment these powerful tools for analyzing hybridization with molecular data, we here present a comprehensive R-package, ClineHelpR, that promotes the genomic cline methodology. The package includes functions that facilitate bgc and Introgress input file generation, output parsing, and functions for outlier-detection and plotting. Locus-wise clinal patterns are visualized by accessing a suite of R-methods that interpret them as a function of the genome-wide average, genomic position along chromosomes, and in relation to spatial and environmental parameters.

## Implementation

### Workflow

**Fig. 1:**
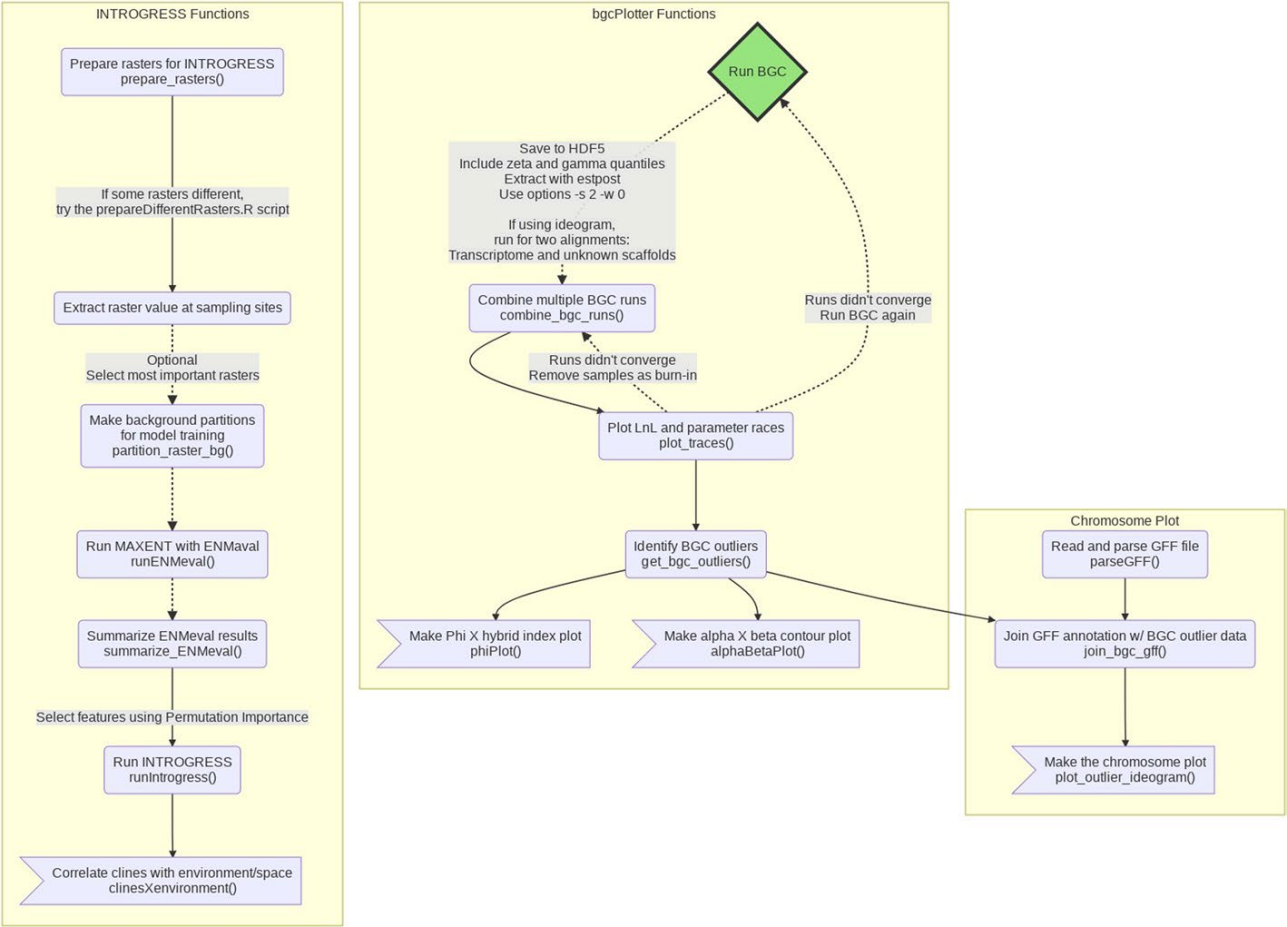
Simplified example workflow listing all available ClineHelpR functions. Yellow boxes group inter-dependent functions working towards producing one or two particular plots (terminal plotting steps depicted as flags). Connecting arrows indicate a pipeline where each step is dependent on the returned R objects. The green ‘Run BGC’ diamond identifies bgc as an external a priori step for the *bgcPlotter* and *chromosome plot* functions. The dotted lines indicate optional steps

The ClineHelpR R-package incorporates an introduction to available functions and can be installed via provided instructions located on the GitHub repository (https://github.com/btmartin721/ClineHelpR). Additionally, we also provide optional Docker [13] integration that allows users to run ClineHelpR from a Docker image with all necessary dependencies and scripts pre-installed in the user’s path. Users can choose to run the Docker container in a command-line terminal or in a Jupyter Notebook. ClineHelpR includes three primary pipelines, a summary of which can be visualized in Fig. 1.

**Fig. 2:**
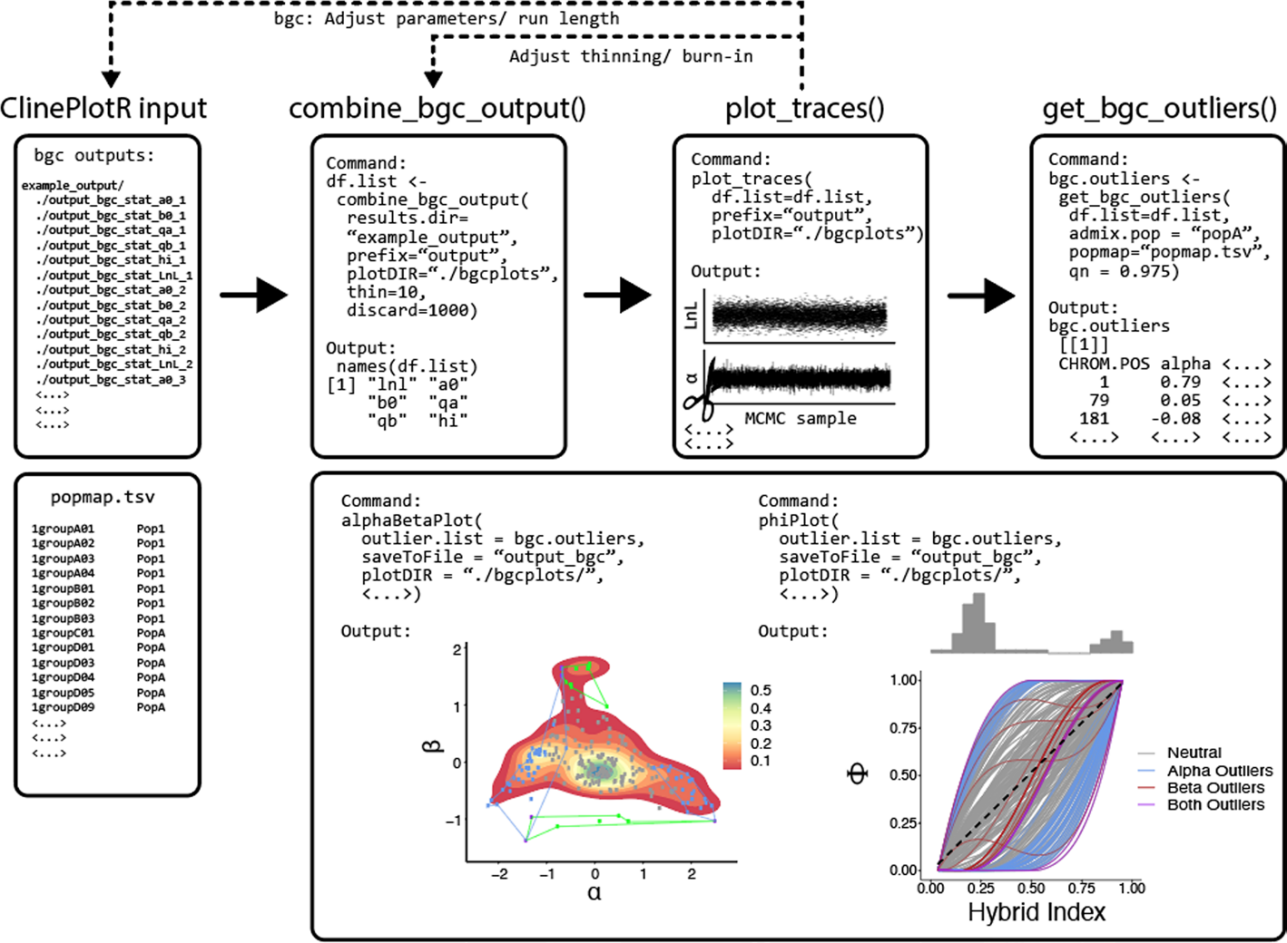
Example workflow for parsing Bayesian genomic cline (bgc) output, visualizing MCMC traces, detecting outliers, and plotting results. The ‘phiPlot’ (right-side, lower right box) shows hybrid indices (x-axis) and probability of parental population1 alleles (y-axis), plus a histogram of hybrid indices in the admixed population. The ‘alphaBetaPlot’ (left-side, lower right box) shows 2D density of cline width/rate representing the cline center (i.e., bias in SNP ancestry; α; x-axis) and steepness of clines (ß; y-axis). Outliers are additionally encapsulated using polygon hulls

The workflow for our bgc pipeline includes functions to aggregate outputs from multiple independent runs, thin MCMC samples, and plot log-likelihood and bgc parameter traces. From these, ClineHelpR can both identify outlier loci using any of several user-defined options and plot locus-wise ancestry probabilities (ϕ) as a function of the hybrid index (Fig. 2). Finally, users can examine the locus-wise relationship between cline center (α) and rate (β), with polygon hulls included to encapsulate 2D ‘outlier space’ for each parameter [14].

**Fig. 3:**
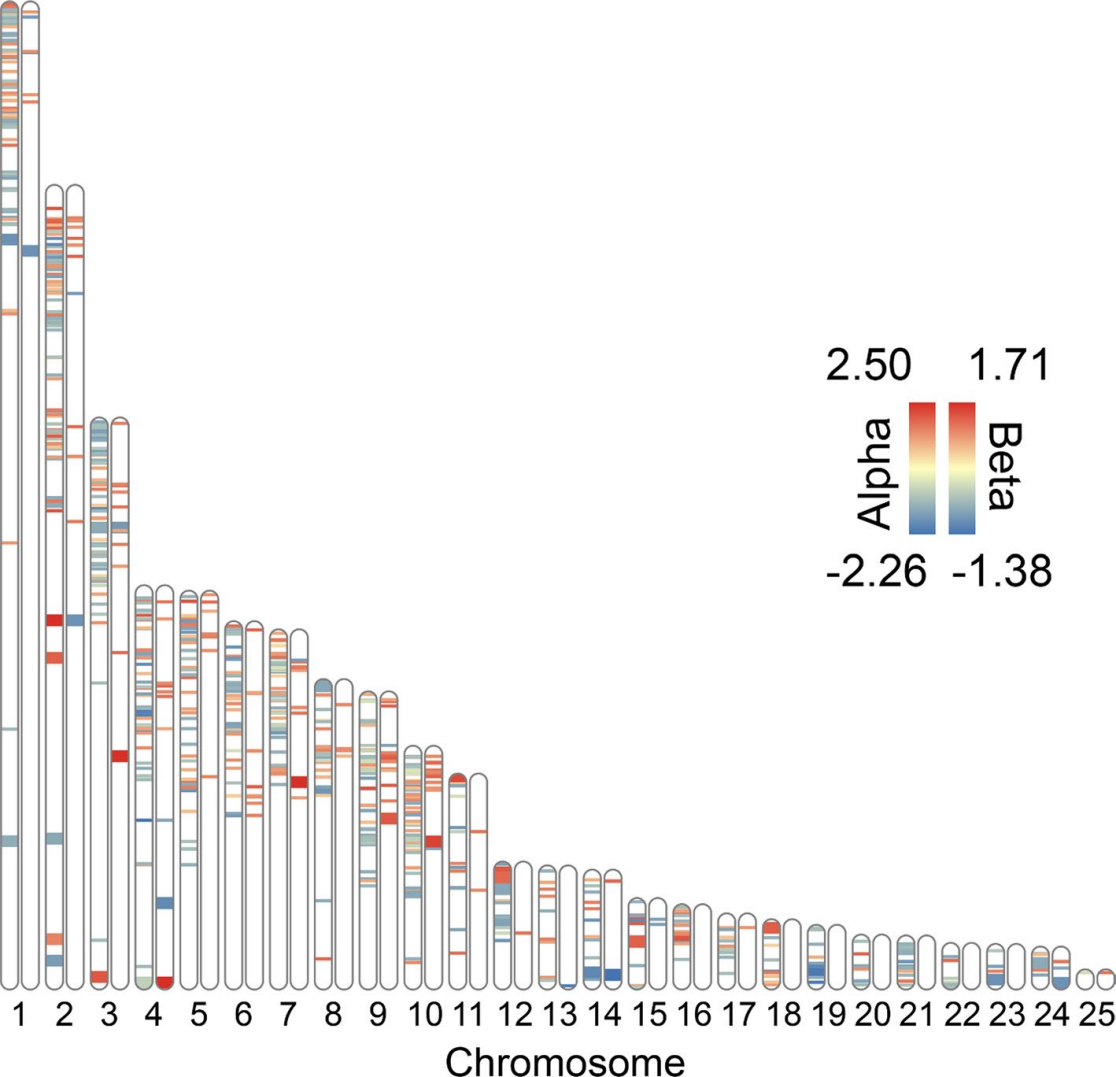
Example ideogram plot using Bayesian genomic cline (bgc) outliers for *Terrapene* ddRAD SNPs (y-axis)*,* plotted onto *Trachemys scripta* chromosomes (x-axis). Chromosomes are duplicated, with alternative heatmaps for cline center (α; left) and rate (ß; right). Larger heatmap bands correspond to SNPs located within known genes, whereas smaller bands were found in unknown scaffolds

ClineHelpR additionally includes accessory functions that allow an examination of variation in clinal parameters across the genome. Although mapping loci to reference assemblies is outside the scope of this package, an example of a workflow using Minimap2 [15] is included in the documentation. If the user has access to physical SNP (single nucleotide polymorphism) coordinates and a closely-related chromosome-level assembly, ClineHelpR can integrate these data with the RIdeogram package [16] to yield karyotype-style ideograms annotated with heatmaps for the bgc cline parameters (Fig. 3).

**Fig. 4:**
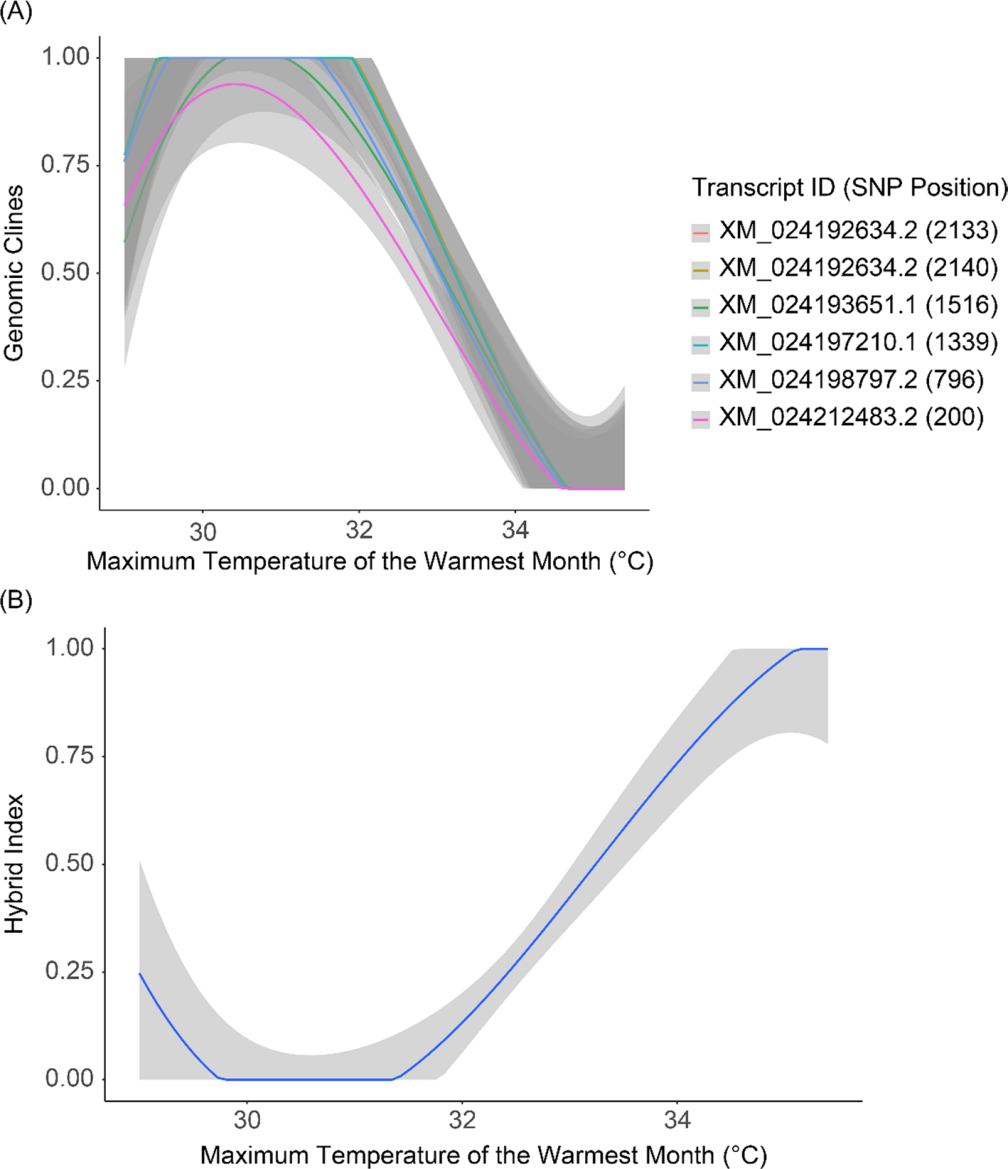
Example plots that can be made using the Introgress pipeline in ClineHelpR. The included climatic variable on the X-axis corresponds to BioClim raster layer 5 (https://worldclim.org). The gray shading indicates confidence intervals for each regression line. **A** Genomic clines for six outlier SNPs mapped to the *Terrapene mexicana triunguis* transcriptome. Transcript IDs correspond to GenBank accession numbers and the position of each SNP (in base pairs) on the locus. **B** Hybrid index output from Introgress *versus* an environmental variable

Functions are also provided to facilitate an Introgress workflow by generating input data frames as well as accessories that embellish the plotting functions already present in Introgress. These accessory functions will visualize spatial patterns (e.g., latitude/longitude) and environmental variables that are inherent to genomic clines (Fig. 4), to include helper functions that invoke ecological niche models (MAXENT: 17 as generated in the R-package ENMeval v2.0 [18, 19]).

### Input and file formats

The primary purpose of ClineHelpR is to simplify the use of software designed to estimate genomic clines. To facilitate this task, ClineHelpR functions and accessory scripts that prepare files for input into bgc and Introgress are available in the GitHub repository and from an external repository https://github.com/tkchafin/scripts, with a few variants. First, ClineHelpR provides native R functions, *genind2bgc* and *genind2introgress* that convert adegenet [20] genind objects to the custom bgc and Introgress formats. These scripts also automatically remove non-biallelic sites and have options to filter both per-site and per-individual missing data at a user-specified threshold and to randomly subsample SNPs. Second, because bgc can additionally consider linkage among loci as well as genotype uncertainty, an input script (*vcf2bgc.py*) that employs the pyVCF Python library (https://pyvcf.readthedocs.io/) is also provided as a means to format ipyrad [21] and stacks [22] VCF (variant call format) files containing annotations for physical position and genotype read counts. Third, the external GitHub repository (see above) contains the *phylip2bgc.pl* script to convert a PHYLIP-formatted alignment containing concatenated SNPs to the custom bgc input format. It can also subset populations and/or individuals from a larger alignment. A similar script, *phylip2introgress.pl*, does likewise with Introgress input. Finally, an additional script in the external repository, *nremover.pl*, is provided to comprehensively filter a PHYLIP-formatted SNP file. The program includes the capacity to filter by matrix occupancy per-individual and per-SNP column, and by minor allele frequency. It can also remove non-biallelic or monomorphic SNPs, and can randomly subsample large datasets. Each of the above scripts are automatically included in the user’s path if using Docker.

### Running bgc and introgress

ClineHelpR also provides functionality to simplify running bgc and Introgress. We supply the *run_bgc.sh* script that runs bgc with the settings specified in the *bgc_settings.txt* file. Once bgc execution is complete, *run_bgc.sh* invokes the *estpost* function to unpack the relevant parameters from the HDF5 output file. and our R API includes the *runIntrogress* function to run Introgress. In both cases, users can adjust a multitude of parameters and settings to suit their needs.

### Outlier detection for Bayesian genomic clines

Output (extracted from HDF5 format using bgc’s *estpost* function) must be named as *prefix*_stat_*param*_*replicate*, where *prefix* is shared across all independent bgc replicates, *param* is an individual output parameter (e.g., LnL), and *replicate* is an integer. Note that the *run_bgc.sh* script handles the output file format automatically. Outputs from any number of replicates can then be parsed, thinned, and combined via the *combine_bgc_output* function in ClineHelpR. The *combine_bgc_output* function provides arguments for the number of MCMC samples to be removed as burn-in, and for a sampling frequency with which to thin samples. Following bgc run aggregation, the MCMC samples can be visually inspected for mixing and convergence using a trace plotting function, *plot_traces*. Adjustments can then be made to thinning or burn-in parameters by re-running the *combine_bgc_output* function or, if necessary, by re-running bgc with altered parameters or increased MCMC length.

A primary goal of genomic cline analysis is to identify loci that possess either excess ancestry or exceptionally steep transitions relative to the genome-wide average. Here, we provide the function *get_bgc_outliers* that offers two outlier detection methods (described in Gompert and Buerkle [9, 10]). Briefly, the first queries if the credibility intervals for the posterior probability distribution of cline parameters *α* or *β* (i.e., cline center and rate, respectively) exclude the neutral expectation (i.e., *α* or *β* = 0). If this interval excludes zero for either parameter, a locus can be flagged as either an *α*-outlier, *β*-outlier, or both.

The second method considers if per-locus parameter estimates are statistically unlikely, given the distribution of values across all loci. This is accomplished by classifying outliers as those for which posterior median α and ß estimates are not encapsulated by the ($\frac{n}{2}$) and ($\frac{1-n}{2}$) quantiles from a conditional α and ß prior distribution (Gaussian with a mean of zero), where *n* represents a user-specified threshold (e.g., 95%, 97.5%). Users can choose whether to classify outliers using any combination of the above methods, but all require the zeta and gamma quantile estimates from the bgc output.

We additionally track whether parameter values are significantly positive or negative. This indicates either an increase (*α* > 0) or decrease (*α* < 0) in the probability of parental population ancestry among hybrids for a given locus, or deviation in the rate of transition in probabilities of locus-specific ancestries towards either very steep (*β* > 0) or wide (*β* < 0) shapes [9].

### Plotting and visualization

We attempted to tailor available visualizations in ClineHelpR towards common applications of Bayesian genomic clines found in the literature, and we will continue to add additional ones as need arises. Many applications seek to identify loci subject to various selective processes [23] by comparing how ancestries transition among loci with respect to the genome wide average. To facilitate this, the *phiPlot* function computes ϕ_ijn_, the probability of parental population1 ancestry for each locus (*i*) and individual (*n*) within each admixed population (*j*) (Eqn. 3 and 4; Gompert and Buerkle [9]). It then produces a plot of ϕ (per locus) on the y-axis against posterior estimates of hybrid index on the x-axis (*sensu* Gompert et al., 2012), with an adjustable color scheme that designates statistical outliers (Fig. 2).

Other applications have specifically examined relationships among cline rate and center parameters [14], and we also do so by implementing the *alphaBetaPlot* function. A 2-D density contour plot of α and ß parameters is produced, with values for individual loci optionally mapped, and with the potential to calculate and plot polygon hulls that encapsulate positive and negative outliers with respect to each parameter (Fig. 2).

### Introgress clines X environment

ClineHelpR also provides functionality to correlate environmental variables with the Introgress genomic clines. The functions *prepare_rasters* and *partition_raster_bg* and are provided to pre-process and prepare a directory of raster files for input into the external package ENMeval v2.0 [19], which runs MAXENT [17]. Processed rasters can then be input into *runENMeval* and *summarize_ENMeval* to run ENMeval and generate numerous summary statistics and plots that can help users deduce the most important environmental variables for their dataset. The environmental variables will then be correlated with Introgress genomic cline outliers using *clinesXenvironment*.

## Results and discussion

Results are depicted and the software validated using a case study examining hybridization between Woodland (*Terrapene carolina carolina*) and Three-toed box turtles (*Terrapene mexicana triunguis*) [3]. Here, we also demonstrate the utility of several additional functions (see Fig. 1) which expand upon the ‘core’ bgc workflow. The first of several can be used to map parameter values of bgc clines onto a chromosomal ideogram via the function *plot_outlier_ideogram* (e.g., Fig. 3). This provides a way to ‘spatially orient’ cline parameters across the genome, in addition to the aforementioned functions for visualizing the relationship among parameters (e.g., Fig. 2).

Briefly, we mapped the *Terrapene* ddRAD sequencing alignment against the available *Terrapene mexicana triunguis* scaffold-level assembly (GenBank Accession: GCA_002925995.2). We then converted the *Terrapene* scaffold coordinates [3] to that of full chromosomes by mapping them against the most closely related full chromosome-level assembly ([24]; GenBank accession: GCA_013100865.1). This was accomplished by employing Minimap2 [15] and PAFScaff (https://github.com/slimsuite/pafscaff). The output from *get_bgc_outliers* and PAFScaff, plus a GFF (general feature format) file read/parsed via the provided functions *parseGFF* and *join_bgc_gff*, were used to plot a heatmap of bgc α- and ß-values on an ideogram. Essentially, the ideogram plot (generated using the RIdeogram R-package) allows the chromosomal locations of each outlier to be visualized (Fig. 3). It also provides a distinction between transcriptomic SNPs falling within known genes *versus* loci from surrounding scaffolds. For additional details, a more in-depth tutorial is provided in https://github.com/btmartin721/ClineHelpR/blob/master/tutorials/ClineHelpR_tutorial_bgc.ipynb.

Other extended functions include a wrapper to simplify running Introgress (*runIntrogress*), and a function that allows genomic clines (Fig. 4A) and hybrid indices (Fig. 4B) from Introgress to be correlated with spatial and environmental variables. To access this functionality, one can run *clinesXenvironment* using the object returned from *runIntrogress* and raster values extracted from each sample locality. Multiple rasters can be included (e.g., the 19 BioClim layers; https://worldclim.org/), and users can run the included ENMeval wrapper functions (*runENMeval* and *summarize_ENMeval*) to identify uninformative layers that may subsequently be excluded from *clinesXenvironment*. These latter functions access MAXENT using the ENMeval pipeline [18], whereby the most informative raster layers are designated with the ‘permutation importance’ statistic.

Genomic clines are useful for assessing patterns of introgression in hybrid zones. Unfortunately, parsing and plotting results from the available genomic cline software require users to write their own scripts. Given that genomic clines have a variety of applications, to include conservation genetics, evolutionary biology, and speciation research, it is clearly important that they be accessible for use by researchers. Here, we present an R-package that automates and greatly simplifies the generation of input and parsing of output from available genomic cline software, as well as the production of publication-quality figures. We also provide extended functionality to explore the effects of environmental and spatial features on genomic clines.

## Conclusions

Essentially, our R-functions automate bgc and Introgress input/output processing and provide several ways of visualizing outlier SNPs across the genome, while also distinguishing known genes and surrounding loci. Furthermore, the extended functionality permits assays of the environmental and spatial effects on genomic clines, enhancing their interpretation and providing greater insight into underlying processes that potentially contribute to the observed patterns. ClineHelpR is intended to be user-friendly, and to this end employs a variety of parameters that can be adjusted to suit specific research needs. In this sense, we also provide Docker and Jupyter Notebook integration to expand the accessibility of our software and facilitate reproducible research. Hopefully, future iterations of genomic cline software can act to extend chromosomal and environmental associations, particularly as whole genome sequencing becomes less expensive and more common.

## Data Availability

Project name: ClineHelpR. Project home page: https://github.com/btmartin721/ClineHelpR. Operating system(s): Platform independent. Programming language: R. Other requirements: R 3.6 or higher; Python 3.6; R-packages: dplyr, bayestestR, scales, reshape2, ggplot2, forcats, gtools, RIdeogram, gdata, adegenet, ENMeval, rJava, raster, sp, dismo, ggforce, concaveman, readr, XML, stringi, devtools, jupyterlab, pyVCF, introgress. License: GNU GPL 3.0. Any restrictions to use by non-academics: No. ClineHelpR is available as a GitHub repository: https://github.com/btmartin721/ClineHelpR. The data used herein is available as an example dataset in an Open Science Framework (OSF) repository [https://doi.org/10.17605/OSF.IO/HBDEP]. A Docker image and Jupyter Notebook tutorials can be found at https://hub.docker.com/repository/docker/btmartin721/clinehelpr and https://github.com/btmartin721/ClineHelpR/tree/master/tutorials, respectively.

## Abbreviations

bgc: Bayesian genomic clines
GFF: general feature format
MCMC: Markov chain Monte Carlo
SNP: single nucleotide polymorphism
VCF: variant call format
